# *Lycorine hydrochloride* Suppresses the Proliferation and Invasion of Esophageal Cancer by Targeting TRIM22 and Inhibiting the JAK2/STAT3 and Erk Pathways

**DOI:** 10.3390/cancers17050718

**Published:** 2025-02-20

**Authors:** Jingyan Liu, Liangxian Qiu, Jialing Chen, Tao Zeng

**Affiliations:** 1 Department of Medical Laboratory, Affiliated Hospital of Guangdong Medical University, Zhanjiang 524023, China; 2 Peking University Shenzhen Hospital, Shenzhen 518036, China

**Keywords:** *Lycorine hydrochloride*, esophageal squamous cell carcinomas, TRIM22, proliferation, metastasis

## Abstract

Background: Tumor metastasis and poor drug efficacy are two of the most common causes of therapeutic failure in cancer patients. The underlying molecular mechanism requires further exploration, and novel effective curative strategies are urgently needed. Nature is a rich source of novel drugs, and *Lycorine hydrochloride* (*Lyc.HCL*) is a natural alkaloid with tremendous therapeutic potential. However, the molecular mechanisms of its antitumor activity are still unknown. In the current study, we investigated the effects and mechanisms of *Lyc.HCL* against esophageal squamous cell carcinomas (ESCCs), which pose serious threats to human life. Methods: An MTS assay and a clone formation assay were used to assess the viability of ESCC cell lines after *Lyc.HCL* treatment in vitro. Apoptosis and cell cycle regulation were analyzed using flow cytometry. Wound healing and Transwell assays were used to analyze cell migration, while invasion was analyzed using the Matrigel Transwell assay. We detected the expression of tripartite motif-containing 22 (TRIM22) through immunohistochemistry and Western blotting. A docking experiment was performed to explore the targets of *Lyc.HCL*. The expression levels of Janus kinase 2 (JAK2)/signal transducer and activator of transcription 3 (STAT3) and phosphoinositide 3-kinase (PI3K)/protein kinase B (AKT)/extracellular signal-regulated kinase (Erk) pathway components were detected through Western blotting. A rescue experiment was performed to determine the potential role of TRIM22. In addition, we explored the in vivo anti-ESCC effects and mechanism of *Lyc.HCL* by using it to treat tumor-bearing mice. Results: The *Lyc.HCL* treatment was found to inhibit esophageal squamous cell carcinoma cell proliferation both in vitro and in vivo by blocking the cell cycle at the G2 phase, inhibiting cell migration and invasion. We found that the TRIM22 protein was highly expressed in ESCCs but not in normal esophageal tissue. *Lyc.HCL* directly targeted TRIM22, decreasing the expression of TRIM22 and the JAK2/STAT3 and Erk signaling pathways, both in vitro and in vivo. Using animal experiments, we observed that the depletion of TRIM22 delayed tumor growth, but this effect was significantly reversed upon TRIM22 overexpression. Conclusions: Taken together, these findings demonstrate that *Lyc.HCL* can effectively suppress ESCC both in vitro and in vivo by targeting TRIM22 and regulating the JAK2/STAT3 and Erk pathways. These results suggest that *Lyc.HCL* may serve as a potential novel therapeutic for ESCC, with TRIM22 emerging as a promising target for treatment.

## Simple Summary

Esophageal squamous cell carcinoma (ESCC) is one of the deadliest cancers due to its ability to metastasize and the lack of effective targets and drugs to treat it. To address this, we explored a natural compound called *Lycorine hydrochloride* (*Lyc.HCL*), derived from a traditional Chinese herb, which has shown promise in fighting cancer. Our study found that *Lyc.HCL* inhibited ESCC cell proliferation, metastasis, and invasion and shrank tumors in mice by directly downregulating TRIM22, a protein that promotes cancer cell growth and metastasis, and interfering with the JAK2/STAT3 and Erk pathways, which are necessary for cancer cell proliferation. These findings suggest that *Lyc.HCL* could be developed into a new, effective treatment for ESCC.

## 1. Introduction

Esophageal cancer is one of the most common and fatal cancers, ranking as the sixth leading cause of cancer-associated deaths globally. Esophageal squamous cell carcinoma (ESCC) is the predominant histological subtype of esophageal cancer, constituting 90% of esophageal cancers worldwide [1]. ESCC is a severe malignancy owing to its aggressive nature and very poor survival rate, with a 5-year survival rate of less than 30% [2]. Although the available screening and curative treatment techniques, such as surgical resection, chemoradiotherapy, and immune therapy, have made remarkable progress, treatment for ESCC is still challenging, and improvement in the prognosis of patients with ESCC remains limited. There is an urgent need to explore promising biomarkers with a higher prognostic accuracy and new drugs for ESCC therapies.

Traditional Chinese medicine is an invaluable source for novel drugs for a variety of diseases due to its extensive history of clinical treatment [3]. *Lycorine* is a pharmacologically active alkaloid extracted from the traditional Chinese medicinal herb *Lycoris radiata* and has significant therapeutic potential. Recent evidence suggests that *Lycorine* and its derivatives have anticancer activities, including significant inhibitory effects on various types of cancers, such as leukemia, lymphoma, melanoma, breast cancer, ovarian cancer, and prostate cancer [4,5]. However, the poor water solubility of *Lycorine* limits its function to some extent [6]. *Lycorine hydrochloride*, the hydrochloride salt form of lycorine, shares a similar structure and displays similar pharmacological effects and could be used as an alternative to lycorine due to its better water solubility [7]. *Lycorine hydrochloride* (*Lyc.HCL*) has been reported to have antitumor functions, including effects against gastric cancer [8] and colon cancer [9], but the effects of *Lyc.HCL* on esophageal squamous cell carcinoma, including its mechanisms and targets, are still unclear. We aimed to determine whether *Lyc.HCL* exerts anticancer effects on ESCC and investigate the underlying mechanisms. Interestingly, data from the GEO datasets show that TRIM22 expression in RBE cells is modulated by *Lyc.HCL* [10], suggesting that TRIM22 may be one of the targets of *Lyc.HCL*. Furthermore, a genome-wide analysis of ESCC indicated that TRIM22 is upregulated in ESCC tissues [11]. This sparked our interest, inspiring us to determine whether *Lyc.HCL* also affects TRIM22 expression in ESCC.

To investigate the role of *Lyc.HCL* in ESCC, we focused on TRIM22, which is a member of the tripartite motif-containing (TRIM) family. TRIM family proteins play key roles in gene regulation, cell growth, signaling, apoptosis, and tumor formation [12], and they are often upregulated or downregulated in cancers, influencing tumor growth and spread [13,14]. This suggests that TRIMs could serve as biomarkers and that the expression levels of TRIMs could serve as prognostic factors for cancers. Additionally, TRIM genes play distinct roles in different cancers. For instance, TRIM22 is downregulated in melanoma, where its reduced expression is associated with cancer progression and poor prognosis [15]. It also serves as a negative regulator of MHCII expression, making it a potential target for checkpoint blockade immunotherapy in cancer treatment [16]. In contrast, TRIM22 is upregulated in glioblastoma, where it promotes tumor proliferation [17], while the upregulation of TRIM22 in osteosarcoma suppresses progression by destabilizing NRF2 and activating the ROS/AMPK/mTOR/autophagy signaling pathway [18]. However, the role of TRIM22 in esophageal squamous cell carcinoma (ESCC) remains unexplored. Our study revealed elevated TRIM22 expression in both ESCC patient samples and various ESCC cell lines. Furthermore, we confirmed that *Lyc.HCL* can effectively suppress TRIM22 expression in these ESCC cell lines. Based on these findings, we hypothesize that *Lyc.HCL* may exert its anti-esophageal squamous cell carcinoma effects through the targeting of TRIM22.

## 2. Materials and Methods

### 2.1. Cell Culture, Reagents, and Treatments

The human esophageal squamous cell carcinoma cell lines used included YES2 (collection: ChemicalBook; catalog number: YS3054), KYSE30 (collection: JCRB; catalog number: JCRB0188), KYSE70 (collection: JCRB; catalog number: JCRB0190), KYSE140 (collection: JCRB; catalog number: JCRB1063), KYSE150 (collection: JCRB; catalog number: JCRB1095), KYSE180 (collection: JCRB; catalog number: JCRB1083), KYSE410 (collection: JCRB; catalog number: JCRB1419), and KYSE450 (collection: JCRB; catalog number: JCRB1430). The reagents (culture medium, fetal bovine serum, and penicillin/streptomycin) and treatment protocol were described previously [19]. *Lycorine hydrochloride* (CAS No. 2188-68-3) was obtained from MedChemExpress. It was dissolved in DMSO and diluted with RPMI 1640 medium before use.

### 2.2. Normal Esophageal Epithelial Tissue, Patient, and Esophageal Squamous Cancer Samples

This study received approval from the Pathology Department of Peking University Shenzhen Hospital (2021-111). From 2018 to 2021, we collected 35 samples of normal esophageal epithelial tissue, 35 samples of peritumoral tissue, and 35 samples of esophageal squamous cancer tissue from the Pathology Department of Peking University Shenzhen Hospital. The normal esophageal epithelial samples were obtained from healthy persons with no history of cancer. The peritumoral tissues were collected from non-cancerous regions of the esophagus, at least 5 cm away from any visible lesions. Esophageal squamous cancer tissues were derived from patients diagnosed with esophageal squamous cell carcinoma, and a tumor tissue sample was obtained from the cancerous region of the esophagus during surgical resection. All procedures involving human participants adhered to the ethical standards of the institution. Informed consent was obtained from all participants. All patients were initially diagnosed with untreated esophageal squamous cancer. We collected and stored all the samples at −80 °C until they were used in immunohistochemistry assays to detect the expression level of TRIM22 protein.

### 2.3. Immunohistochemistry

The cancer tissue and normal tissue samples from patients were treated with xylene, followed by a graded alcohol treatment. Antigen retrieval was then performed in a 0.01 M citrate buffer. Hydrogen peroxide was used for blocking. The tissue sections were incubated with goat serum for 20 min. The slides were then incubated overnight at 4 °C with a TRIM22 antibody (1:300; Proteintech, Rosemont, IL, USA). Immunostaining of TRIM22 was performed using the EliVision Super Kit from Maixin (Fuzhou, China). Two independent pathologists randomly examined all the tumor slides. TRIM22 staining was observed in both the cytoplasmic and nuclear compartments of the tumor cells. ESCC tissue was considered TRIM22-positive if the cells were stained brown (due to the DAB chromogen) and the staining was localized predominantly in the cytoplasm and nucleus of the cells.

### 2.4. Cell Proliferation and Apoptosis Assay

The cell proliferation and apoptosis assays were conducted as previously described in [19] and were used to evaluate cell growth and programmed cell death.

### 2.5. Colony Formation Assay

The colony formation assay was conducted as previously described in [19], enabling the assessment of cell proliferation and the cells’ colony-forming ability. ESCC cells were seeded at a density of 5000 cells per well in 6-well plates. The cells were cultured in RPMI 1640 medium supplemented with 10% fetal bovine serum and 1% penicillin/streptomycin; 0 μmol/mL, 2 μmol/mL, 4 μmol/mL, or 6 μmol/mL *Lyc.HCL* was added to the different groups. The medium was not replaced for 2 weeks to allow the colonies to form naturally under the given conditions. After the 2-week incubation period, the cultures were washed with pre-cooled PBS, fixed with methanol, and stained with a 0.1% crystal violet solution for 30 min. The colonies were automatically examined and quantified using Image-Pro Plus.

### 2.6. Cell Cycle Assay

The cell cycle assay was performed, following a previously established protocol [19], to assess the distribution of cells across the different phases of the cell cycle.

### 2.7. Cell Migration Assay

The cell migration assay was performed following a previously published protocol [19].

### 2.8. Transwell Migration Assays

The migration of the cells was evaluated using Transwell cell culture chambers with 6.5 mm diameter polycarbonate membrane filters with a pore size of 8 μm (Neuro Probe, Gaithersburg, MD, USA). The upper chamber was filled with 200 μL of serum-free medium with different concentrations of Lyc.HCL and 5 × 10^5^ YES2 or KYSE150 cells, and the lower chamber was filled with 500 μL of medium containing 20% FBS. After 24 h of incubation at 37 °C, the non-migrated cells were removed from the upper surface of the membrane with a cotton swab. The filters were then fixed in methanol for 10 min, stained with a crystal violet solution for 1 h, and counted. Five random microscopic fields (100×) were counted per well, and the mean value was analyzed.

### 2.9. Cell Invasion Assay

A Matrigel invasion assay was conducted to examine tumor cell invasion. Initially, the Transwell upper chambers were filled with 0.1 mL of Matrigel (Becton Dickinson, Bedford, MA, USA) and incubated at 37 °C for 1 h. Subsequently, the cells were treated with varying concentrations of *Lyc.HCL* medium for 24 h. The cells were then trypsinized and suspended in serum-free RPMI 1640 medium at a final concentration of 5 × 10^5^ cells/mL. The subsequent steps were carried out as previously described [19].

### 2.10. Lentiviral Infection, Transient Transfection, and Cas9/sgRNA KO

The plasmids and siRNAs were transfected into cells using the Lipofectamine 3000 reagent (Invitrogen, Grand Island, NY, USA). The experiment was conducted strictly following the instructions provided by the transfection reagent manufacturer. The Leti-Cas9-puromycin and single-guide RNA (sgRNAs) lentiviruses were designed and constructed by Genechem (Shanghai, China). Lentiviruses carrying Cas9 and sgRNAs were used to infect YES2 and KYSE150 cells. After 48 h of infection, the cells were cultured in fresh medium containing puromycin for 15 days to screen for cells with stable expression. All plasmids were designed and constructed by Hanbio (Shanghai, China).

### 2.11. Docking Experiment

The docking experiment was carried out with Discovery Studio 2017 R2. Three dimensional structures of Lyc.HCL were downloaded from PubChem Compound. The structure of TRIM22 was obtained from AlphaFold. The receptor preparation included removing water molecules and impurity ions and adding polar hydrogen atoms. The predicted binding energy (kcal mol^−1^) was calculated. The most reasonable complex structures were identified according to binding energy and selected as the initial models for the subsequent simulations. The docking of the ligand to the receptor in CDOCKER Experiment was performed using software Discovery Studio 2017 R2. The highest scoring ligand receptor binding pose was selected, and the RMSD was obtained.

### 2.12. Western Blotting

Protein extraction and Western blotting were conducted as described previously [19]. Briefly, proteins were isolated using RIPA lysis buffer (Beyotime, Shanghai, China). Then, 24 μg of protein was loaded and separated using 12% SDS–polyacrylamide gel electrophoresis and transferred onto a polyvinylidene difluoride membrane and incubated with JAK2 (#3230T), PI3K (#17366), mTOR (#2983), AKT (#4691), p-AKT (#4060T), Erk (#4370), p-Erk (#4370T), and GAPDH (#51332) antibodies, purchased from Cell Signaling Technology and diluted at a ratio of 1:1000; TRIM22 (#13744-1-AP) antibody, purchased from Proteintech and diluted at a ratio of 1:500; and STAT3 (#ab68153) and p-STAT3 (#ab267373) antibodies, purchased from Abcam and diluted at a ratio of 1:1000. All antibodies were diluted using a diluent purchased from Beyotime Biotechnology. The chemiluminescence signals were detected using an Amersham Imager 600 (GE, New York, NY, USA).

### 2.13. Rescue Assays

YES2 and KYSE150 cells were seeded at a concentration of 20,000 cells/well in 6-well plates and incubated overnight. TRIM22^siRNA^ and a plasmid for expressing TRIM22 were transiently transfected into the YES2 and KYSE150 cells for 24 h, and then *Lyc.HCL* (4 μmol/mL) was added to the treated cells (referred to as YES2^shTRIM22^, YES2^TRIM22^, KYSE150^shTRIM22^, and KYSE150^TRIM22^) for 48 h. Then, the cells were collected and analyzed using colony formation, cell cycle, cell migration, cell invasion, and Western blotting assays, as previously described.

### 2.14. Tumor Xenograft Experiments

Six-week-old female BALB/c nude mice were randomly divided into nine groups, with six mice in each group. The groups were named as follows: KYSE150-NC, KYSE150-NC-*Lyc.HCL* (5 mg/kg), KYSE150-NC-*Lyc.HCL* (10 mg/kg), KYSE150-shTRIM22, KYSE150-shTRIM22-*Lyc.HCL* (5 mg/kg), KYSE150-shTRIM22-*Lyc.HCL* (10 mg/kg), KYSE150-TRIM22, KYSE150-TRIM22-*Lyc.HCL* (5 mg/kg), and KYSE150-TRIM22-*Lyc.HCL* (10 mg/kg). Each group received a subcutaneous injection of 5 × 10^6^ cells into the right rear of the mice. After seven days of subcutaneous tumorigenesis, the mice in the groups KYSE150-NC-*Lyc.HCL* (5 mg/kg), KYSE150-NC-*Lyc.HCL* (10 mg/kg), KYSE150-shTRIM22-*Lyc.HCL* (5 mg/kg), KYSE150-shTRIM22-*Lyc.HCL* (10 mg/kg), KYSE150-TRIM22-*Lyc.HCL* (5 mg/kg), and KYSE150-TRIM22-*Lyc.HCL* (10 mg/kg) were treated with an intraperitoneal injection of *Lyc.HCL* (5 mg/kg twice a day per mouse) and *Lyc.HCL* (10 mg/kg twice a day per mouse) for 14 days, while the other two groups were injected with DMSO. The tumor volumes (mm^3^) and body weight were measured twice a day using a vernier caliper and a scale and calculated using the formula tumor volume = L × W × 0.5 (L: the longest diameter of the tumor; W: the shortest diameter of the tumor).

Subsequently, the mice were euthanized using an intraperitoneal injection of 150 mg/kg sodium pentobarbital to halt their breathing, and cardiac arrest was confirmed. The tumor tissue was then dissected and photographed. The tumor tissue was embedded, cut into 4 µm thick slices, fixed in 4% paraformaldehyde for 15 min at room temperature, and then stained with hematoxylin for 10 min and eosin for 2 min at room temperature. The tissue was observed under an Olympus BX40 light microscope (Olympus Corporation, Nottingham, MD, USA). An immunohistochemistry assay was performed to analyze the expression of TIRM22, JAK2, and p-AKT. A Western blot analysis was used to quantify the expression of TRIM22 and JAK2/STAT3 and Erk pathway components in the tumor tissues.

### 2.15. Statistical Analysis

The experiments were conducted in duplicate and repeated three times. A statistical analysis of the in vitro data was performed using Student’s *t*-test and one-way ANOVA. Student’s *t*-test was used to compare the different groups. A *p*-value of less than 0.05 was considered statistically significant for all data (* *p* < 0.05, ** *p* < 0.01, and *** *p* < 0.001).

## 3. Results

### 3.1. TRIM22 Is Upregulated in ESCC Patient Tissues and Various Human ESCC Cell Lines (YES2, KYSE30, KYSE70, KYSE140, KYSE180, KYSE410, and KYSE450 Cells)

**Figure 1:**
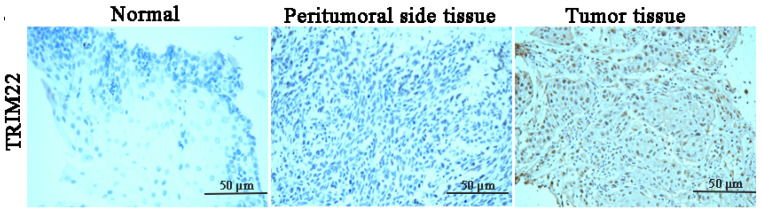
High expression of TRIM22 in esophageal squamous cell carcinoma patient specimens. Immunohistochemistry assay shows the expression of TRIM22 in normal esophageal tissue, peritumoral tissue, and esophageal cancer tissue.

TRIM22 protein expression was examined in 35 normal esophageal tissue specimens, 35 peritumoral tissue specimens, and 35 ESCC specimens using immunohistochemistry. The number of cells expressing TRIM22 is summarized in Table 1. The ESCC specimens were collected from untreated patients who were newly diagnosed, and TRIM22 expression was observed exclusively in ESCC tissue cells. Consistently, in normal esophageal and peritumoral tissues, negative TRIM22 staining was observed. In contrast, positive TRIM22 staining was evident in both the nuclear and cytoplasmic compartments of esophageal tumor cells (Figure 1, consistent with previous reports) and was classified as positive staining [20]. In the ESCC tissues, high TRIM22 expression was observed in all cases, with both nuclear and cytoplasmic localization. Additionally, we detected TRIM22 expression in the human esophageal squamous cell carcinoma cell lines. The Western blot analysis demonstrated significantly elevated TRIM22 protein levels in YES2, KYSE30, KYSE70, KYSE140, KYSE150, KYSE180, KYSE410, and KYSE450 cells, with particularly high expression in YES2 and KYSE150 cells (Supplementary Materials Figure S1A,B).

### 3.2. Lyc.HCL Inhibits the Proliferation of ESCC Cells

**Figure 2:**
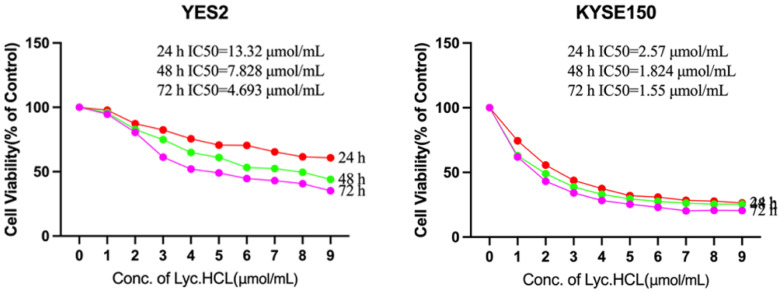
Effect of *Lyc.HCL* on proliferation of human esophageal squamous cell carcinoma (ESCC) cell lines. YES2 and KYSE150 cells were treated with the indicated concentrations of *Lyc.HCL* for 24 h, 48 h, and 72 h. Cell viability was assessed using the MTS assay. IC50 values were calculated using GraphPad Prism 5.0 software. Data are presented as mean ± SD.

Eight ESCC cell lines (YES2, KYSE30, KYSE70, KYSE140, KYSE150, KYSE180, KYSE410, and KYSE450) were treated with *Lyc.HCL* at concentrations of 0, 1, 2, 3, 4, 5, 6, 7, 8, and 9 μmol/mL for 24 h, 48 h, and 72 h. Cell viability was measured using the MTS assay, and the inhibitory effect was quantified by determining the 50% inhibitory concentration (IC50). All tested cell lines exhibited dose- and time-dependent sensitivities to the *Lyc.HCL* treatment (Figure 2 and Supplementary Materials Figure S2). Notably, the cell lines showed varying degrees of sensitivity to the treatment, with KYSE150 and KYSE180 displaying higher sensitivities. At 72 h, the IC50 values were 1.55 μmol/mL for KYSE150 cells (Figure 2) and 1.254 μmol/mL for KYSE180 cells (Supplementary Materials Figure S2), consistent with previous reports indicating that the IC50 value for *Lyc.HCL* typically does not exceed 7.5 μmol/mL [4]. Based on these results and the TRIM22 expression profile in ESCC cell lines (Supplementary Materials Figure S1A), we selected YES2 and KYSE150 cells, which have higher TRIM22 expression, for further experiments.

**Figure 3:**
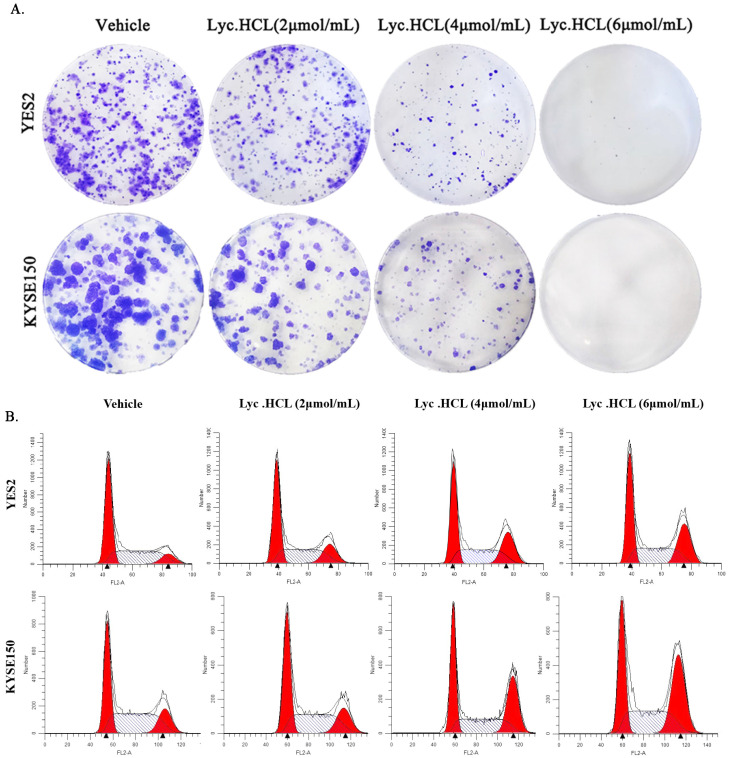
Effect of *Lyc.HCL* on cell colony formation and cell cycle of ESCC cells. (**A**) Colony formation assay results for YES2 and KYSE150 cells. ESCC cells were treated with the indicated concentrations of *Lyc.HCL* for 14 days. (**B**) YES2 and KYSE150 cells were treated with vehicle, 2, 4, and 6 µmol/mL of *Lyc.HCL* for 48 h, and then stained with PI and subjected to FACS; the black triangle indicates the specific phases of cell cycle.

Next, a cell colony formation assay was performed using YES2 and KYSE150 cells to assess the effects of the *Lyc.HCL* treatment on the cell population and its proliferation capacity. The cells were seeded into 6-well plates and incubated for 2 weeks. The results of the colony formation assay are shown in Figure 3A and Supplementary Materials Figure S3A. A reduction in colony numbers was observed with increasing *Lyc.HCL* concentration. In conclusion, both the MTS and colony formation assays demonstrated that the *Lyc.HCL* treatment significantly inhibited ESCC cell proliferation and colony formation in a dose-dependent manner.

### 3.3. Lyc.HCL Induces Cell Cycle Arrest in ESCC Cells

After confirming the positive effects of *Lyc.HCL* on the proliferation of YES2 and KYSE150 cell lines, we investigated the molecular mechanisms underlying its inhibitory effect on ESCC cells. We examined the impact of the *Lyc.HCL* treatment on the cell cycle and found that it induced cell cycle arrest at the G2/M phase in both YES2 and KYSE150 cells (Figure 3B). The data were analyzed using ModFit 5.0; the results are presented in Supplementary Materials Figure S3B,C.

### 3.4. Lyc.HCL Inhibits the Metastasis and Invasion of YES2 and KYSE150 Cells

**Figure 4:**
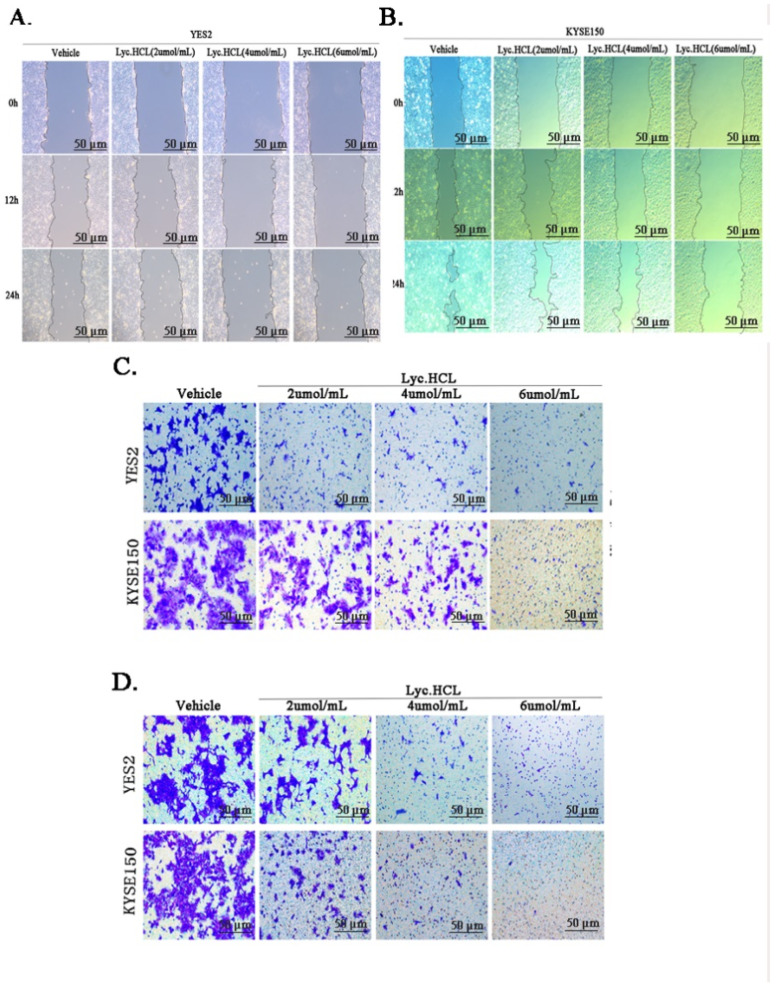
*Lyc.HCL* inhibited migration and invasion of YES2 and KYSE150 cells. (**A**,**B**) YES2 and KYSE150 cells were treated with vehicle, 2, 4, and 6 µmol/mL *Lyc.HCL* for 48 h. Cell migration was evaluated using the wound healing assay. Scale bar: 200 µm. (**C**) YES2 and KYSE150 cells, pretreated with vehicle, 2, 4, and 6 µmol/mL of *Lyc.HCL* for 12 h, were plated onto the apical side of the filters in serum-free medium containing either vehicle or *Lyc.HCL*. Medium containing 20% FBS was placed in the basolateral chamber for 24 h to act as a chemoattractant. Cells on the bottom of the filter were stained using 0.5% crystal violet and then counted. (**D**) Before the experiment, the Transwell chamber was covered with matrix glue. YES2 and KYSE150 cells, pretreated with vehicle, 2, 4, and 6 µmol/mL *Lyc.HCL* for 12 h, were plated onto the apical side of the filters in serum-free medium containing either vehicle or *Lyc.HCL*. Medium containing 20% FBS was placed in the basolateral chamber for 24 h to act as a chemoattractant. Cells on the bottom of the filter were stained using 0.5% crystal violet and then counted.

After confirming the critical role of *Lyc.HCL* in regulating cell cycle arrest in ESCC cells, we further investigated its inhibitory effects on the migration and invasion of YES2 and KYSE150 cells. To assess cell migration, a scratch wound (200 μm in width) was created on a confluent monolayer. Migration into the scratched area was observed, and wound closure was quantified after 0, 12, and 24 h of the *Lyc.HCL* treatment. Figure 4A,B and Supplementary Materials Figure S4A,B show that the *Lyc.HCL* treatment significantly reduced the migration of YES2 and KYSE150 cells into the scratched zone in a concentration-dependent manner, indicating the effective inhibition of cell motility. Additionally, cell migration was also assessed using the Transwell assay (Figure 4C), which showed that the *Lyc.HCL* treatment inhibited the migration of YES2 and KYSE150 cells in a dose-dependent manner. ImageJ and GraphPad Prism 5.0 were used for the data analysis (Supplementary Materials Figure S4C). In addition, a Matrigel invasion assay was conducted to evaluate the effect of *Lyc.HCL* on cell invasion in YES2 and KYSE150 cells. The results were consistent with those from the wound closure and Transwell assays, indicating that *Lyc.HCL* effectively suppressed cell invasion in a dose-dependent manner (Figure 4D and Supplementary Materials Figure S4D).

### 3.5. Lyc.HCL Targets TRIM22 and Regulates Its Expression in YES2 and KYSE150 Cells

**Figure 5:**
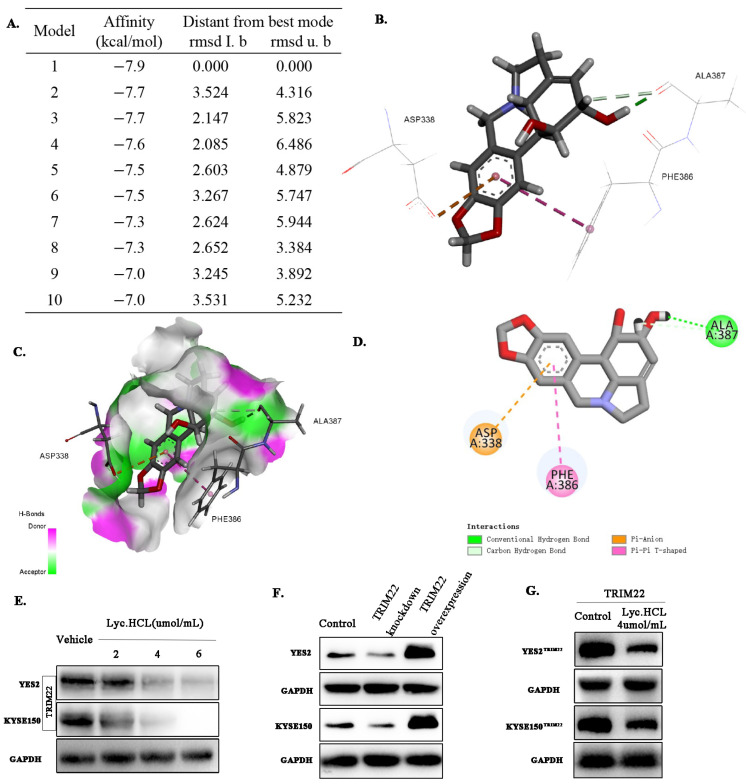
TRIM22 expression in YES2 and KYSE150 cells can be regulated by *Lyc.HCL*. (**A**–**D**) Docking model of *Lyc.HCL* with TRIM22. (**A**) Docking affinity and binding poses of *Lyc.HCL* with TRIM22. (**B**) Interaction pattern of *Lyc.HCL* with the residues of TRIM22. (**C**) *Lyc.HCL* binding with the pocket through hydrogen bonds, where pink represents hydrogen bond donors and green represents hydrogen bond acceptors. (**D**) Two-dimensional diagram of receptor and ligand. (**E**) Western blotting was performed to assess TRIM22 expression in YES2 and KYSE150 cells treated with vehicle or *Lyc.HCL* at concentrations of 2, 4, and 6 µmol/mL for 48 h. (**F**) YES2 and KYSE150 cells were transfected with either a TRIM22 plasmid or siRNA to overexpress or knockdown TRIM22, respectively. (**G**) *Lyc.HCL* treatment reduced the overexpression of TRIM22 in YES2^TRIM22^ and KYSE150^TRIM22^ cells.

TRIM22 has been implicated in promoting tumorigenesis and cancer progression [21,22], and previous reports suggested that *Lyc.HCL* modulates TRIM22 expression [10]. Our study confirmed that TRIM22 is highly expressed in both ESCC patient samples and cell lines. Based on these findings, we hypothesized that *Lyc.HCL* inhibits ESCC by targeting TRIM22. To investigate our hypothesis, molecular docking experiments were performed using *Lyc.HCL* and the crystal structure of TRIM22, which were analyzed with Discovery Studio 2017 R2. Figure 5A presents the docking affinity and binding poses of *Lyc.HCL* with TRIM22. The top-ranked binding model exhibited an affinity of −7.9 kcal/mol, with its RMSD values indicating high stability and accuracy of the docking prediction. Figure 5B–D illustrate the interaction details of the docking model with the highest affinity. In this model, *Lyc.HCL* forms conventional hydrogen bonds and a carbon–hydrogen bond with ALA387 of TRIM22, as well as a Pi–anion interaction at ASP338 and a Pi–Pi T-shaped interaction at PHE386. To further visualize the interaction, the binding site was represented in a surface format (Figure 5C), highlighting the spatial arrangement and interaction contributions. These results strongly support the conclusion that *Lyc.HCL* directly binds to TRIM22.

We then explored how *Lyc.HCL* regulates TRIM22 expression. Western blotting was performed on ESCC cells, and the results demonstrated that the *Lyc.HCL* treatment significantly suppressed TRIM22 expression in both YES2 and KYSE150 cell lines in a dose-dependent manner (Figure 5E). The quantification of TRIM22 expression in YES2 and KYSE150 cells is shown in Supplementary Materials Figure S5A. Further confirmation was obtained by constructing stable cell lines (YES2 and KYSE150) that either overexpressed or knocked down TRIM22 (Figure 5F and Supplementary Materials Figure S5B). As illustrated in Figure 5G, the *Lyc.HCL* treatment reduced TRIM22 expression in both YES2^TRIM22^ and KYSE150^TRIM22^ cells. The quantification of relative TRIM22 expression is presented in Supplementary Materials Figure S5C.

### 3.6. Lyc.HCL Exerts Its Anticancer Effect in ESCC Cells by Targeting TRIM22

Additionally, to investigate whether *Lyc.HCL* exerts its antitumor effect on ESCC cells by targeting TRIM22, we constructed transient cell models of TRIM22 overexpression and knockdown that were then treated with *Lyc.HCL*.

**Figure 6:**
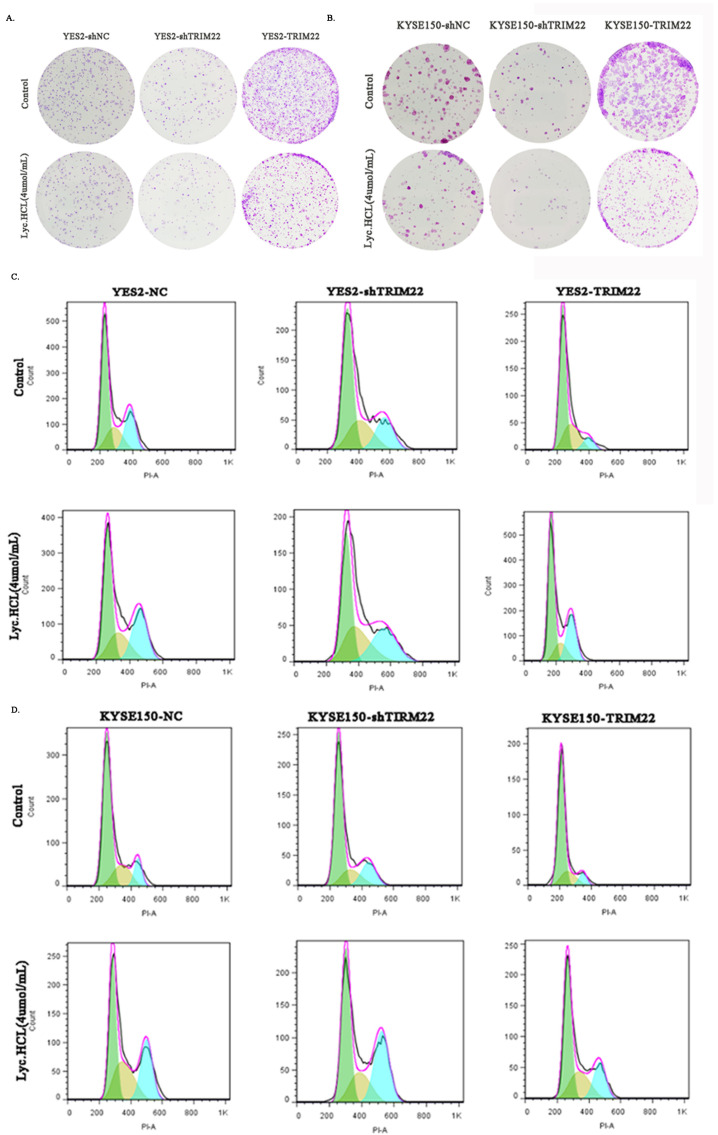
*Lyc.HCL* exerted an antiproliferative effect on ESCC cells by targeting TRIM22 through regulating the cell cycle. (**A**,**B**) Representative images showing that TRIM22 overexpression alleviated *Lyc.HCL*-induced inhibition of colony formation, whereas shTRIM22 promoted *Lyc.HCL*-induced colony formation inhibition. (**C**,**D**) Cell cycle assays revealed that TRIM22 overexpression reduced *Lyc.HCL*-induced cell cycle arrest, while shTRIM22 increased *Lyc.HCL*-induced cell cycle arrest (green for G1, blue for G2, and yellow for S phase).

Cell proliferation was assessed using a colony formation assay. TRIM22 knockdown inhibited cell growth, and *Lyc.HCL* further reduced cell growth in YES2^shTRIM22^ and KYSE150^shTRIM22^ cells. In contrast, TRIM22 overexpression promoted cell growth, and *Lyc.HCL* also inhibited the growth of YES2^TRIM22^ and KYSE150^TRIM22^ cells (Figure 6A,B and Supplementary Materials Figure S6A,B). To explore the underlying molecular mechanisms of the *Lyc.HCL*-induced inhibition of ESCC cell proliferation, a cell cycle assay was performed. Overexpression of TRIM22 in YES2^TRIM22^ and KYSE150^TRIM22^ cells alleviated cell cycle arrest, whereas the *Lyc.HCL* treatment reversed this alleviation. Conversely, TRIM22 knockdown in YES2^shTRIM22^ and KYSE150^shTRIM22^ cells increased cell cycle arrest at the G2/M phase, and *Lyc.HCL* further promoted G2/M arrest in these cells (Figure 6C,D and Supplementary Materials Figure S6C,D). These findings suggest that by regulating the cell cycle, TRIM22 overexpression mitigates the cell growth inhibition induced by *Lyc.HCL*, whereas TRIM22 knockdown enhances the antiproliferative effects of *Lyc.HCL*.

**Figure 7:**
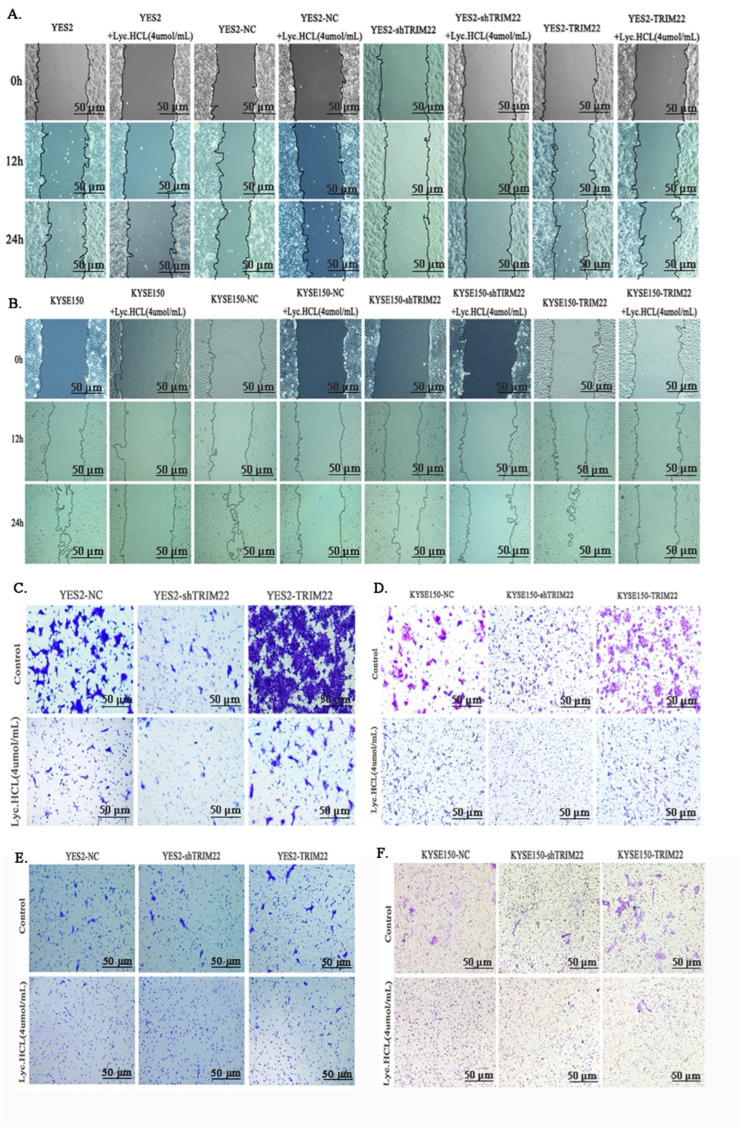
TRIM22 overexpression promotes cell migration and invasion, while TRIM22 knockdown reduces these abilities. (**A**,**B**) Wound healing assay showing the migration of ESCC^TRIM22^ cells, ESCC^shTRIM22^ cells, and *Lyc.HCL*-treated cells. (**C**,**D**) Transwell migration assay assessing the migration ability of ESCC^TRIM22^, ESCC ^shTRIM22^, and *Lyc.HCL*-treated cells. (**E**,**F**) Transwell invasion assay evaluating the invasive potential of ESCC^TRIM22^, ESCC^shTRIM22^, and *Lyc.HCL*-treated cells. Before the experiment, the Transwell chamber was coated with Matrigel. Cells that invaded through the membrane were stained with 0.5% crystal violet and counted.

To investigate the role of TRIM22 in the *Lyc.HCL*-induced inhibition of migration and invasion of cells, we conducted wound healing, Transwell, and cell invasion assays. As shown in Figure 7 and Supplementary Materials Figure S7, TRIM22 overexpression significantly enhanced cell migration and invasion, while the *Lyc.HCL* treatment inhibited the cell motility induced by TRIM22 overexpression. In contrast, TRIM22 knockdown reduced cell migration and invasion, and *Lyc.HCL* further diminished these effects. These results suggest that overexpression of TRIM22 alleviates the inhibition of cell motility induced by *Lyc.HCL*, while TRIM22 knockdown enhances the suppression of cell migration mediated by *Lyc.HCL*.

### 3.7. Lyc.HCL Targets TRIM22 and Has a Potential Anti-Esophageal Cancer Effect Through the Regulation of the JAK2/STAT3 and Erk Pathways

**Figure 8:**
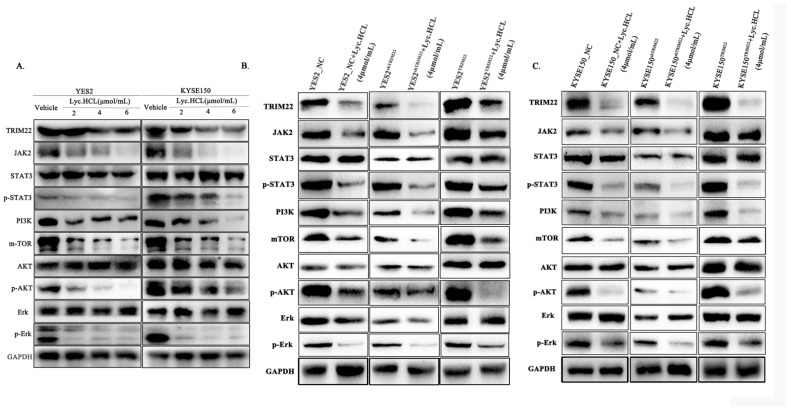
*Lyc.HCL* suppresses the JAK2/STAT3 and ERK signaling pathways by targeting TRIM22 in ESCC. The expression levels of THIM22, JAK2, STAT3/p-STAT3, PI3K, mTOR, AKT/p-AKT, and Erk/p-Erk were evaluated using the corresponding antibodies and Western blotting. All the experiments were independently repeated at least three times. (**A**) YES2 and KYSE150 cells were treated with the indicated concentrations of *Lyc.HCL* for 48 h, and the expression levels of the JAK2/STAT3 and ERK pathway components were assessed by Western blotting. (**B**,**C**) The Western blot results showed changes in the expression of TRIM22, JAK2, p-STAT3, PI3K, mTOR, p-AKT, and p-Erk in YES2, KYSE150, YES2^TRIM22^, YES2^shTRIM2^2, KYSE150^TRIM22^, KYSE150^shTRIM22^, and *Lyc.HCL*-treated cells.

We demonstrated that *Lyc.HCL* inhibits the proliferation, metastasis, and invasion of ESCC by targeting TRIM22. To further explore the underlying mechanisms, we investigated the associated signaling pathways. Previous studies have shown that TRIM family proteins regulate colorectal cancer cell proliferation, migration, and invasion through the JAK2/STAT3 pathway [23]. Based on this, we hypothesized that TRIM22 may similarly regulate ESCC proliferation, metastasis, and invasion via the JAK2/STAT3 pathway, which is also involved in the TRIM22-mediated inhibition of viral replication [24]. In ESCC cells, we observed that the *Lyc.HCL* treatment downregulated both JAK2/STAT3 and ERK signaling pathway components in a dose-dependent manner (Figure 8A). The quantification of the relative protein expression in YES2 and KYSE150 cells was performed using ImageJ (Supplementary Materials Figure S8A).

**Figure 9:**
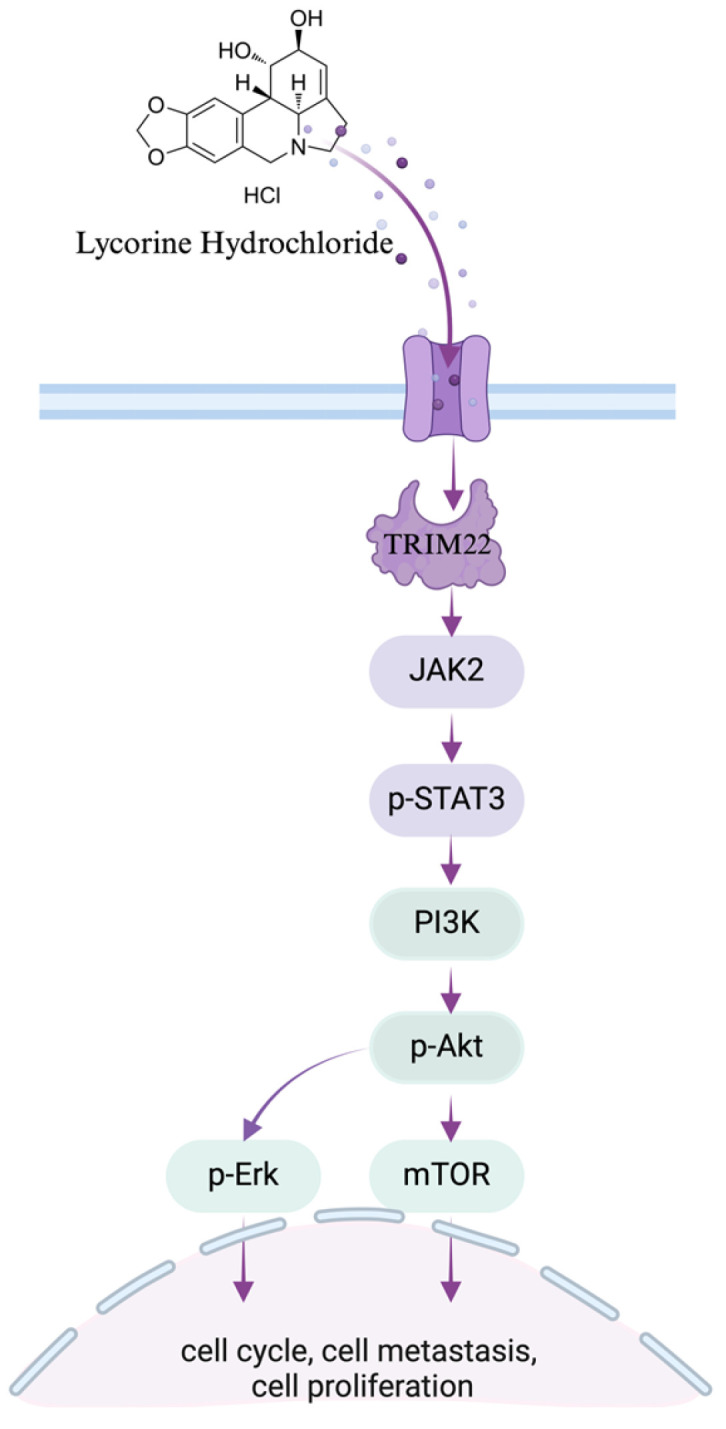
Schematic diagram of the TRIM22-JAK2/STAT3 and ERK signaling pathway axis.

Next, we investigated the JAK2/STAT3 and ERK signaling pathways in YES2^TRIM22^, YES2^shTRIM22^, KYSE150^TRIM22^, and KYSE150^shTRIM22^ cells, both with and without *Lyc.HCL* treatment. As shown in Figure 8B,C and Supplementary Materials Figure S8B,C, TRIM22 overexpression promoted the activation of the JAK2/STAT3 and ERK pathways, whereas the *Lyc.HCL* treatment suppressed the TRIM22 overexpression-induced pathway activation. In contrast, TRIM22 knockdown reduced the expression of JAK2/STAT3 and ERK signaling pathway components, and the *Lyc.HCL* treatment further enhanced the inhibition of these pathways in TRIM22-knockdown cells. Furthermore, combining the *Lyc.HCL* treatment with TRIM22 knockdown resulted in more pronounced inhibition of the JAK2/STAT3 and ERK pathways than either treatment alone. These findings suggest that *Lyc.HCL* exerts its anti-ESCC effects by targeting TRIM22 and regulating the JAK2/STAT3 and ERK signaling pathways (Figure 9).

### 3.8. Lyc.HCL Alleviates Tumor Growth by Targeting TRIM22 In Vivo

**Figure 10:**
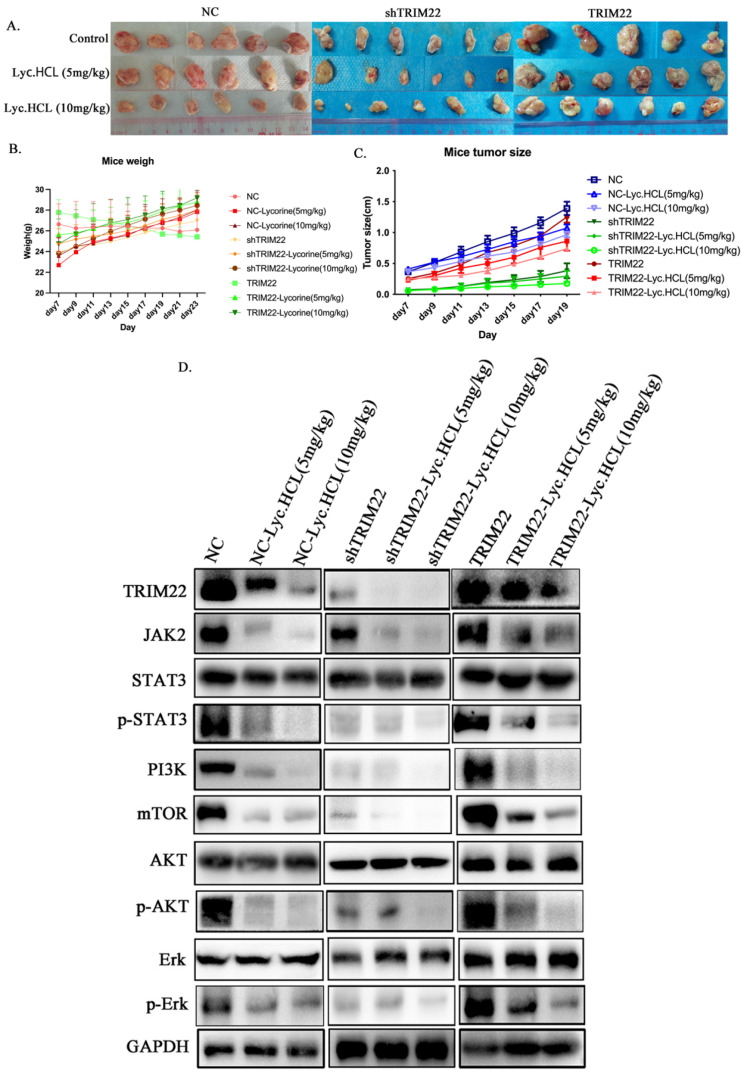
*Lyc.HCL* alleviates tumor growth by targeting TRIM22 and regulating the JAK2/STAT3 and ERK pathways in vivo. KYSE150 control, KYSE150^shTRIM22^, and KYSE150^TRIM22^ cells were subcutaneously injected into nude mice. Seven days later, the mice were intraperitoneally injected with either DMSO or *Lyc.HCL* (5 or 10 mg/kg, twice a day) until day 23. (**A**) Tumor tissues were harvested after the mice were euthanized on day 23. (**B**) The body weights of the nude mice were recorded throughout the treatment period. (**C**) The in vivo tumor sizes were measured using a vernier caliper during the treatment. (**D**) A Western blot analysis was performed to assess the expression of JAK2/STAT3 and ERK pathway components in tumor tissues dissected from the different treatment groups.

*Lyc.HCL* was previously reported to suppress tumorigenesis in bladder cancer in vivo [25]. To further investigate the antitumor effects and potential targets of *Lyc.HCL* in ESCC, we established a subcutaneous tumor xenograft model. The results shown in Figure 10A,C show that TRIM22 overexpression significantly increased tumor size, while the *Lyc.HCL* treatment notably inhibited the tumor growth induced by TRIM22. Additionally, TRIM22 knockdown reduced tumor formation and size, and *Lyc.HCL* further promoted tumor size reduction in TRIM22-knockdown tumors. Supplementary Materials Figure S9D–F show the analysis of tumor size in the mouse model. Figure 10B illustrates the changes in the mice’s body weights, which reflect the tumor-induced cachexia to some extent (a heavier body weight correlates with less severe cachexia). Based on the results (Figure 10B) and the analysis (Supplementary Materials Figure S9A–C), we concluded that TRIM22 overexpression exacerbated cachexia, while *Lyc.HCL* alleviated the cachexia induced by TRIM22 overexpression. In contrast, TRIM22 knockdown reduced cachexia, and treatment of TRIM22-knockdown tumors with *Lyc.HCL* further promoted a decrease in cachexia.

**Figure 11:**
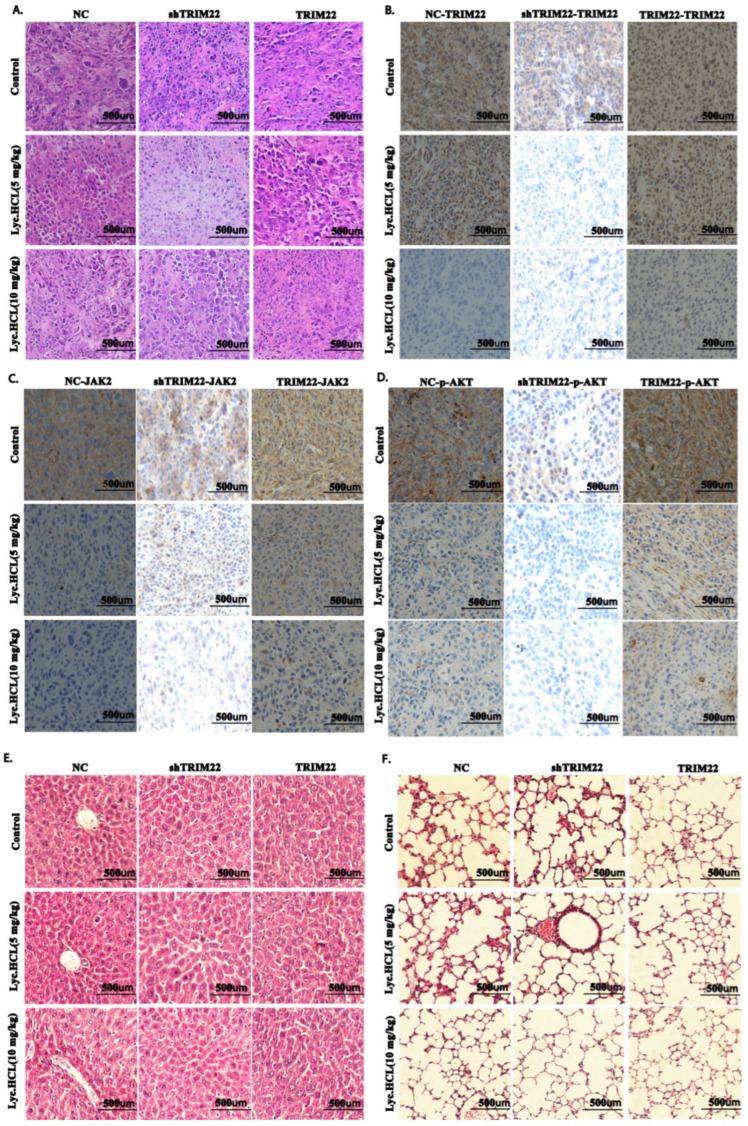
HE and IHC analysis of tissues from the different groups. (**A**) Representative HE images of dissected tumor tissues from the different groups. Scale bar: 500 μm. IHC analysis of (**B**) TRIM22, (**C**) JAK2, and (**D**) p-AKT expression in tumor tissues from the different groups. Scale bar: 500 μm. Representative HE images of dissected (**E**) liver and (**F**) lung tissues from the different groups. Scale bar: 500 μm.

The subsequent Western blot analysis of tumor tissues revealed that *Lyc.HCL* significantly inhibited TRIM22 expression, as well as the expression of JAK2/STAT3 and ERK signaling pathway components. Additionally, *Lyc.HCL* alleviated the overexpression of JAK2/STAT3 and ERK pathway components that were induced by TRIM22 overexpression, while enhancing the inhibition of these proteins in TRIM22-knockdown tumors (Figure 10D). Supplementary Materials Figure S9G–P show the quantification of the relative protein expression presented in Figure 10D. H&E staining was conducted to evaluate the tumor morphology under the different treatment conditions and TRIM22 expression modifications (Figure 11A). Tumors in the NC group exhibited a dense cellular architecture with frequent tumor cell nuclear division. TRIM22 overexpression increased the tumor cell density and the tumors displayed an atypical morphology, whereas TRIM22 knockdown (shTRIM22) resulted in a more organized and less aggressive tumor morphology. Treatment with *Lyc.HCL* (5 mg/kg and 10 mg/kg) significantly reduced the cellular density and frequency of tumor cell nuclear division, highlighting its potential antitumor effects. Immunohistochemical staining was performed to evaluate the effects of the *Lyc.HCL* treatment on the expression of TRIM22, JAK2, and p-AKT, key proteins in the JAK2/STAT3 signaling pathway. Robust TRIM22 expression was observed in the TRIM22-overexpressing group, while shTRIM22 tumors exhibited markedly reduced expression. The *Lyc.HCL* treatment significantly suppressed TRIM22 expression (Figure 11B). JAK2 expression followed a similar trend: it was suppressed in the shTRIM22 group and elevated in the TRIM22-overexpressing group. However, treatment with *Lyc.HCL*, particularly at 10 mg/kg, led to a notable reduction in JAK2 expression (Figure 11C). Consistent trends were observed for the p-AKT staining (Figure 11D). TRIM22 knockdown reduced p-AKT levels, while overexpression enhanced them. The *Lyc.HCL* treatment further suppressed p-AKT expression, underscoring its potential regulatory role in TRIM22-mediated signaling. To assess the potential toxicity of *Lyc.HCL*, we also examined the morphology of liver and lung tissues. The liver sections from all the groups appeared to retain normal histology (Figure 11E), and the lung tissue analysis revealed normal alveolar structures in all groups (Figure 11F), suggesting that *Lyc.HCL* has minimal toxicity, which is consistent with the findings of previous reports [26].

## 4. Discussion

Esophageal squamous cell carcinoma (ESCC) remains one of the most lethal cancers, with a five-year survival rate of less than 30% [27]. It is imperative to identify more effective therapeutic targets and develop new drugs to enhance the treatment efficiency for ESCC. Emerging evidence suggests that *Lyc.HCL* has great potential in the treatment of human cancers [28]. However, the precise antitumor mechanisms and targets of *Lyc.HCL* in ESCC remain poorly understood. In this study, we investigated the anticancer effects of *Lyc.HCL* on ESCC and explored its molecular targets. Our findings indicate that *Lyc.HCL* can significantly inhibit cell proliferation, migration, and invasion in multiple ESCC cell lines, including YES2 and KYSE150. Notably, we identified TRIM22, a member of the TRIM family of proteins, as a critical target of *Lyc.HCL*. TRIM22 has previously been implicated in cancer progression [29], and our results showed that *Lyc.HCL* can downregulate TRIM22 expression in a dose-dependent manner. This, in turn, leads to the suppression of key signaling pathways involved in ESCC progression, such as the JAK2/STAT3 and ERK pathways. The JAK2/STAT3 and ERK pathways are well known for their roles in regulating cell proliferation, survival, migration, and invasion in various cancers [30]. Our data suggest that *Lyc.HCL* exerts its anticancer effects through the inhibition of these critical signaling cascades, which are activated by TRIM22. Importantly, we further confirmed the therapeutic potential of *Lyc.HCL* in vivo by using a xenograft model. The *Lyc.HCL* treatment delayed tumor growth and alleviated tumor-induced cachexia, demonstrating its in vivo efficacy. Histological analyses of tumor tissues using IHC staining revealed that *Lyc.HCL* effectively reduced the expression of TRIM22 and other key signaling proteins such as JAK2 and p-AKT, further supporting its role in modulating these pathways. Additionally, the H&E staining analysis of liver and lung tissues showed no significant signs of toxicity, suggesting that *Lyc.HCL* has a favorable safety profile, which is crucial for its clinical application. These results highlight the potential of *Lyc.HCL* as a novel therapeutic agent for ESCC. By targeting TRIM22 and modulating the JAK2/STAT3 and ERK signaling pathways, *Lyc.HCL* may offer a promising strategy for the treatment of ESCC and other cancers in which TRIM22 plays a significant role.

In this study, we explored the anti-ESCC mechanisms of action of *Lyc.HCL*. A key finding was the involvement of TRIM22, a protein previously implicated in the regulation of cellular differentiation and proliferation in various cancers [31]. We observed that TRIM22 was highly expressed in both ESCC patient tissues and ESCC cell lines (Figure 1 and Supplementary Materials Figure S1). This elevated expression of TRIM22 in ESCC is consistent with findings in glioblastoma, suggesting a potential role for TRIM22 in the pathogenesis of both cancers [32]. However, the precise functional role and expression of TRIM22 in human cancers remain somewhat ambiguous. For example, in melanoma patients, lower TRIM22 expression is correlated with shorter survival times [15], whereas in non-small-cell lung cancer, TRIM22 overexpression is associated with poorer survival outcomes [21]. These contradictory findings highlight the complex role of TRIM22 in different cancer types. In our study, we found that overexpression of TRIM22 promoted ESCC cell proliferation, migration, and invasion, which suggests that TRIM22 functions as an oncogenic driver in ESCC. This is further supported by our observation that TRIM22 knockdown suppressed cell proliferation and migration, indicating that TRIM22 could be a promising therapeutic target for ESCC. Interestingly, the *Lyc.HCL* treatment inhibited TRIM22 expression in ESCC cell lines, specifically in YES2 and KYSE150 cells. This led us to hypothesize that *Lyc.HCL* might exert its anti-ESCC effects by targeting TRIM22. To investigate this hypothesis further, we performed molecular docking studies and rescue assays using TRIM22-overexpression and -knockdown cell lines. The results confirmed that *Lyc.HCL* directly binds to TRIM22, supporting the idea that TRIM22 is a functional target of *Lyc.HCL*. Moreover, we observed that *Lyc.HCL* inhibited the cell proliferation, migration, and invasion induced by TRIM22 overexpression. Conversely, *Lyc.HCL* enhanced the inhibition of these processes in TRIM22-knockdown cells. These findings suggest that TRIM22 acts as an oncogenic protein in ESCC and may serve as a key target for *Lyc.HCL* in treating ESCC. However, it is also important to note that *Lyc.HCL* inhibited ESCC cell proliferation, migration, and invasion even when TRIM22 was knocked down. This suggests that *Lyc.HCL* may exert its anticancer effects through multiple targets, highlighting the possibility of additional, yet unidentified, molecular targets for *Lyc.HCL*. This multi-target activity could be a crucial factor in the efficacy of *Lyc.HCL* against ESCC.

Uncontrolled tumor growth is commonly associated with dysregulated cell cycle progression [33]. To better understand the molecular mechanisms underlying the effects of *Lyc.HCL* on ESCC, we investigated its impact on the cell cycle. Our results showed that Lyc.HCL caused a significant block in the cell cycle at the G2/M phase, ultimately leading to inhibition of cell proliferation. *Lyc.HCL* not only inhibited the cell cycle progression induced by TRIM22 overexpression, but it also enhanced cell cycle arrest when TRIM22 was knocked down. These findings support the hypothesis that *Lyc.HCL* induces cell cycle arrest at the G2/M phase through targeting TRIM22. Cell cycle regulation is tightly linked to several key signaling pathways, including the JAK2/STAT3 pathway, which has been identified as a critical regulator of cell cycle progression and proliferation in many cancers [34,35,36]. Notably, TRIM family proteins, including TRIM22, have been shown to modulate JAK2/STAT3 signaling [16,37]. Our results suggest that *Lyc.HCL*’s inhibition of ESCC cell proliferation is, at least in part, mediated through the regulation of the JAK2/STAT3 pathway by targeting TRIM22. This provides further evidence that TRIM22 serves as an important mediator of tumor progression in ESCC. In addition to the JAK2/STAT3 pathway, the PI3K/AKT/mTOR pathway, which lies downstream of JAK2/STAT3 signaling, plays a central role in the regulation of a wide range of cellular processes, including survival, growth, and angiogenesis [38]. This pathway is often hyperactivated in cancer, driving tumorigenesis. The PI3K/AKT pathway is also known to collaborate with the ERK signaling pathway, another key regulator of tumor proliferation, invasion, and metastasis [38,39]. Our findings suggest that *Lyc.HCL* may inhibit ESCC cell proliferation by simultaneously targeting both the JAK2/STAT3 and ERK pathways, underscoring its potential as a multi-target therapeutic agent for ESCC.

Tumor metastasis and invasion are major contributors to poor prognosis in cancer patients and play a critical role in determining treatment outcomes [40]. In this study, we demonstrated that *Lyc.HCL* can effectively inhibit the metastasis and invasion of the ESCC cell lines YES2 and KYSE150 in a dose-dependent manner. Overexpression of TRIM22 significantly enhanced cell migration and invasion, while knockdown of TRIM22 resulted in a marked reduction in these processes. Importantly, treatment with *Lyc.HCL* reversed the migratory and invasive phenotypes induced by TRIM22 overexpression, suggesting that *Lyc.HCL* exerts its anti-metastatic and anti-invasive effects by targeting TRIM22 in ESCC. The molecular mechanisms underlying these effects were further explored by focusing on the JAK2/STAT3 signaling axis, which has been shown to play a critical role in tumor cell migration, invasion, and metastasis [41,42]. The PI3K/AKT/mTOR pathway, a downstream effector of JAK2/STAT3, has been widely implicated in regulating various aspects of tumor progression, including migration and invasion [43,44,45]; this provides additional support for the idea that drugs targeting the JAK2/STAT3 axis may offer promising therapeutic strategies for inhibiting tumor metastasis and invasion. Moreover, the ERK pathway, which is activated during various stages of cancer metastasis, is another known regulator of tumor cell migration and invasion [46,47,48,49,50,51]. Our study further corroborated these observations by showing that *Lyc.HCL* suppresses both the JAK2/STAT3 and ERK pathways, leading to reduced migration and invasion of ESCC cells. This suggests that *Lyc.HCL* exerts its anti-metastatic and anti-invasive effects through a multi-target approach, effectively controlling several key pathways involved in tumor progression. While our findings indicate that *Lyc.HCL* modulates critical signaling pathways to inhibit tumor metastasis and invasion, further investigation is needed to explore the role of downstream molecules, such as extracellular matrix (ECM) components and proteins involved in epithelial-to-mesenchymal transition (EMT), which are essential steps in metastasis and invasion [52]. Understanding how *Lyc.HCL* affects these molecular events will provide a more comprehensive view of its anti-metastatic and anti-invasive mechanisms, potentially leading to the development of more effective therapeutic strategies for ESCC and other malignancies.

Currently, no small-molecule compounds specifically targeting TRIM22 are being studied in clinical trials. The efforts to modulate TRIM family proteins, including TRIM22, have largely focused on their E3 ubiquitin ligase activity and their involvement in key signaling pathways. Indirect strategies, such as targeting upstream modulators or associated signaling pathways like JAK/STAT3, have been explored as potential therapeutic strategies. In this paper, we present the first evidence demonstrating the potential of *Lyc.HCL* as a potent inhibitor of ESCC cell proliferation, metastasis, and invasion. *Lyc.HCL* represents a novel therapeutic modality for ESCC that has not been previously explored in depth, providing an exciting new avenue for intervention in this aggressive cancer type. Moreover, our study is the first to propose TRIM22 as a promising target for therapeutic interventions in ESCC. This discovery could serve as a pioneering step toward the development of TRIM22-targeted therapies, marking a significant advancement in the field. By highlighting the potential of *Lyc.HCL* to target TRIM22 and modulate key signaling pathways such as JAK2/STAT3 and Erk, we present a comprehensive mechanism of action that could be leveraged for therapeutic benefit. This study not only bridges basic research with therapeutic development, but it also offers a potential new, effective treatment strategy for ESCC. The identification of *Lyc.HCL* as a modulator of TRIM22 provides a solid foundation for exploring small molecules that could directly target TRIM22. Future studies should focus on identifying and developing specific TRIM22-targeting compounds to assess their therapeutic potential and further advance the field.

While our study provides valuable insights into the effects of *Lyc.HCL* on ESCC, several limitations should be addressed in future research to fully understand its therapeutic potential. Firstly, our focus was solely on TRIM22 expression in ESCC patients who had not received any treatment or drug intervention. It is essential to incorporate more detailed clinical data, including tumor stage, metastasis status, and patient age and gender, to assess the potential of TRIM22 as a prognostic biomarker for ESCC. This broader clinical context could enhance the understanding of TRIM22’s role in ESCC progression and its relevance to treatment outcomes. Secondly, while we demonstrated that *Lyc.HCL* inhibits cancer cell growth, metastasis, and invasiveness by targeting TRIM22 and modulating the JAK2/STAT3 and ERK pathways in vitro and in vivo, our in vivo experiments were limited to a subcutaneous xenograft model. To comprehensively evaluate the anti-metastatic and anti-invasive effects of *Lyc.HCL*, further in vivo studies using models that better reflect human disease progression—such as metastasis models or organ-specific models—are needed. Thirdly, we used human ESCC cell line-derived xenograft models for our in vivo studies, which may not fully recapitulate the heterogeneity and complexity of human tumors. Patient-derived xenograft (PDX) models or transgenic mice, which preserve the genetic and histological features of human ESCC more accurately, would provide more reliable data and could help validate the therapeutic effects of *Lyc.HCL* in a more clinically relevant context. Lastly, while our findings indicate that *Lyc.HCL* exerts its anticancer effects through the modulation of TRIM22 and its downstream signaling pathways, the specific downstream mechanisms through which *Lyc.HCL* inhibits the cell cycle, metastasis, and invasion of ESCC remain to be fully elucidated.

## 5. Conclusions

In summary, *Lyc.HCL*, a monomer derived from a traditional Chinese medicinal herb, demonstrates potent anti-ESCC effects. *Lyc.HCL* holds promise as a potential therapeutic agent for ESCC as it targets TRIM22, a critical molecule, and modulates the JAK2/STAT3 and ERK signaling pathways. Our findings show that TRIM22 is upregulated in ESCC tissues and cell lines, where it plays a significant role in cell proliferation, metastasis, and invasion. Additionally, the JAK2/STAT3 and ERK pathways are likely involved in the TRIM22-mediated regulation of these processes. These results suggest that TRIM22 may serve as both a diagnostic biomarker and a therapeutic target for ESCC.

## Figures and Tables

**Table 1:** Number of cells expressing TRIM22 in different tissues.

Tissue No.	Normal Tissue	Peritumoral Tissue	ESCC Tissue
1	0	0	1556
2	0	0	1507
3	0	0	1687
4	0	0	3657
5	0	0	3676
6	0	0	2031
7	0	0	2240
8	0	0	1786
9	0	0	1099
10	0	0	2345
11	0	0	1455
12	0	0	2345
13	0	0	1596
14	0	0	2329
15	0	0	2020
16	0	0	1945
17	0	0	1425
18	0	0	1709
19	0	0	2063
20	0	0	1113
21	0	0	1162
22	0	0	1092
23	0	0	1927
24	0	0	1634
25	0	0	1552
26	0	0	3374
27	0	0	3068
28	0	0	2813
29	0	0	1552
30	0	0	1142
31	0	0	1750
32	0	0	1414
33	0	9	1213
34	0	0	1709
35	0	0	1786

## Data Availability

The data used to support the findings of this study are available from the corresponding author upon request.

## Supplementary Materials

The following supporting information can be downloaded at: https://www.mdpi.com/article/10.3390/cancers17050718/s1, Figure S1. High expression of TRIM22 in human esophageal squamous cell carcinoma cell lines. Figure S2. Effect of *Lyc.HCL* on proliferation of human esophageal squamous cell carcinoma cell lines (ESCC). Figure S3. Data analysis of cell colony formation and cell cycle of ESCC cells. Figure S4. Data analysis of *Lyc.HCL* inhibited migration and invasion in YES2 and KYSE150 cells. Figure S5. Data analysis of Figure 5E–G. Figure S6. Data analysis of Figure 6. Figure S7. Data analysis of Figure 7. Figure S8. Data analysis of Figure 8. Figure S9 Data analysis of Figure 10.

## Abbreviations

ESCC	esophageal squamous cell carcinoma
*Lyc.HCL*	lycorine hydrochloride
TRIM	tripartite motif-containing
TCM	traditional Chinese medicine
JAK2	Janus kinase 2
STAT3	signal transducer and activator of transcription 3
PI3K	phosphoinositide 3-kinase
AKT	protein kinase B
ERK	extracellular signal-regulated kinase
ECM	extracellular matrix
EMT	epithelial-to-mesenchymal transition
