# Supplementary material for: Lycorine hydrochloride Suppresses the Proliferation and Invasion of Esophageal Cancer by Targeting TRIM22 and Inhibiting the JAK2/STAT3 and Erk Pathways
Source: Cancers (Basel). 2025 Feb 20;17(5):718. doi: 10.3390/cancers17050718 (PMC11898953; doi:10.3390/cancers17050718)
Supplement: Supplementary file 1 [file cancers-17-00718-s001.zip › cancers-3325857-supplementary.pdf]

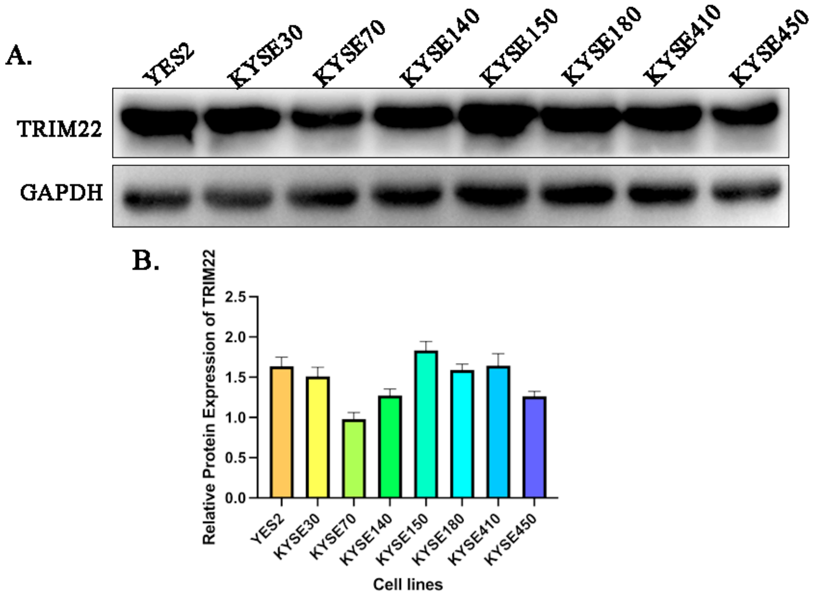

Supplementary Figure S1. High expression of TRIM22 in human esophageal squamous cell carcinoma cell lines. (A) Western blotting was performed to detect TRIM22 expression in human esophageal squamous cell carcinoma cell lines. (B) Quantification of relative TRIM22 protein expression in different cell lines was performed using ImageJ.

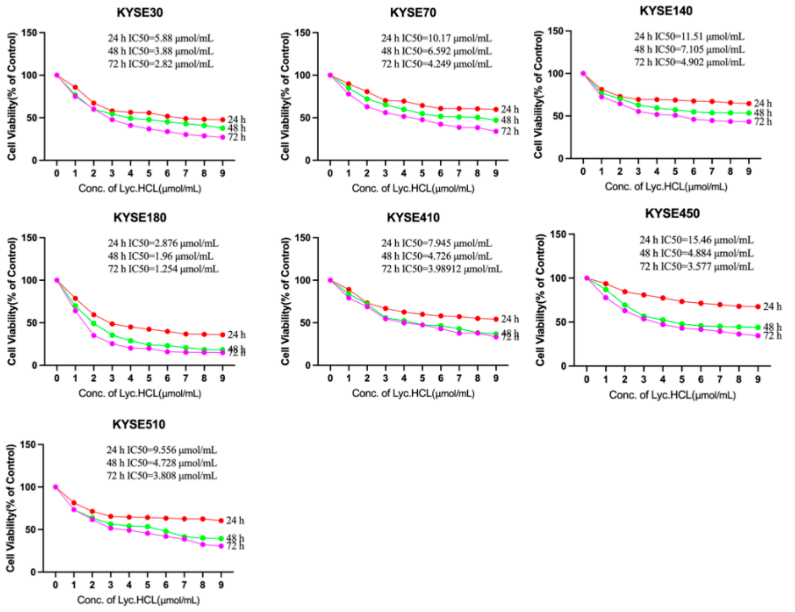

Supplementary Figure S2. Effect of *Lyc.HCL* on proliferation of human esophageal squamous cell carcinoma cell lines (ESCC). KYSE30, KYSE70, KYSE140, KYSE180, KYSE410 and KYSE450 cells were treated with the indicated concentrations of *Lyc.HCL* for 24h, 48h and 72 h, cell viability was assessed using MTS assay. IC50 values were calculated using the GraphPad Prism 5.0 software. Data are presented as mean ± SD.

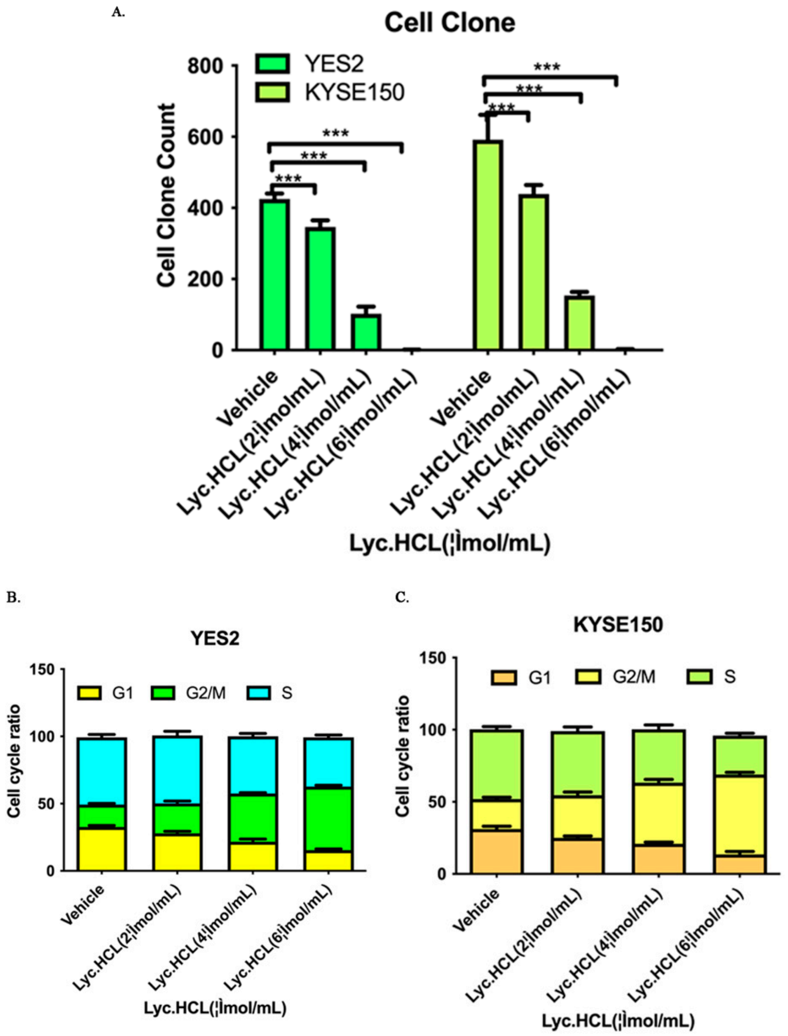

Supplementary Figure S3. Data analysis of cell colony formation and cell cycle of ESCC cells. (A) The quantification of the cell colonies in Figure 3A is presented as the mean percentage of viable cells (mean ± SD), averaged from 3 independent experiments, each with 3 replicates per condition. (B and C) cell cycle was analyzed by Mod Fit 5.0. All data are presented as the mean ± SD. A one-way analysis of variance, followed by a Tukey's post-hoc test, was used to compare the different groups. *$P<0.05$, **$P<0.01$, ***$P<0.001$ versus vehicle.

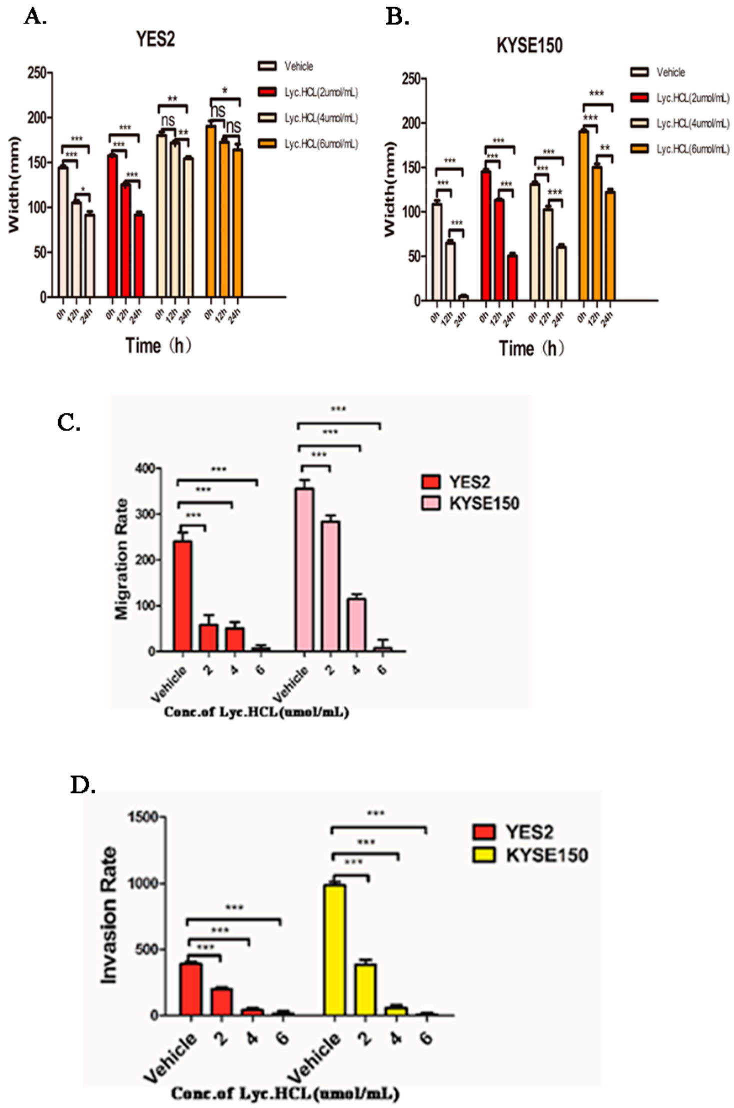

Supplementary Figure S4. Data analysis of *Lyc.HCL* inhibited migration and invasion in YES2 and KYSE150 cells. (A and B) Gap Width data of Figure 4A and 4B were presented as the mean ± SD. A one-way analysis of variance, followed by a Tukey's post-hoc test, was used to compare the different groups. *P<0.05, **P<0.01, ***P<0.001 versus vehicle. (C) Quantification of the migrated cells in Figure 4C was displayed on the right. The results were displayed as the mean ± SD. A one-way analysis of variance, followed by a Tukey's post-hoc test, was used to compare the different groups. *P<0.05, **P<0.01, ***P<0.001 versus vehicle. (D) Quantification of the invasive cells in Figure 4D was displayed on the right. The results were displayed as the mean ± SD. A one-way analysis of variance, followed by a Tukey's post-hoc test, was used to compare the different groups. *P<0.05, **P<0.01, ***P<0.001 versus vehicle.

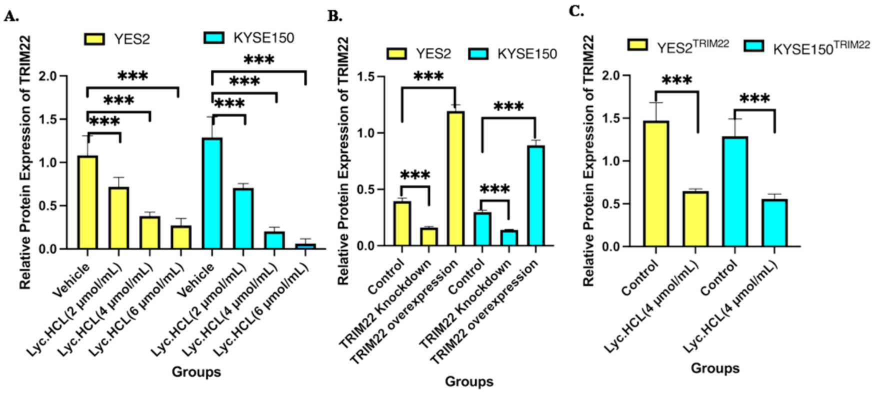

Supplementary Figure S5. Data analysis of Figure 5E, 5F and 5G. (A) The quantification of relative TRIM22 protein expression in Figure 5E was performed using ImageJ. (B) The quantification of relative TRIM22 protein expression in Figure 5F was performed using Image J. (C) Relative protein expression of TRIM22 in Figure 5G was quantified using Image J.

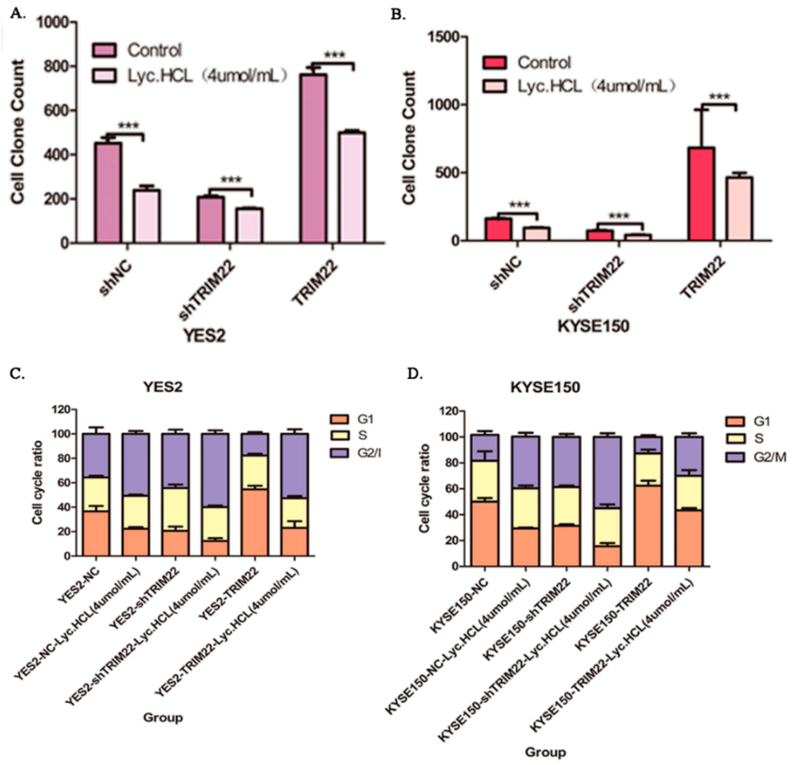

Supplementary Figure S6. Data analysis of Figure 6. (A and B) Quantification of cell colonies in Figure 6A and 6B, expressed as the mean percentage of viable cells (mean ± SD), averaged from three independent experiments, each with three replicates per condition. (C and D) Cell cycle analysis of Figure 6C and 6D were performed using FlowJo 10.9.0. All data are presented as mean ± SD. Statistical significance was determined using a one-way analysis of variance (ANOVA), followed by Tukey's post-hoc test. *P < 0.05, **P < 0.01, ***P < 0.001 versus vehicle.

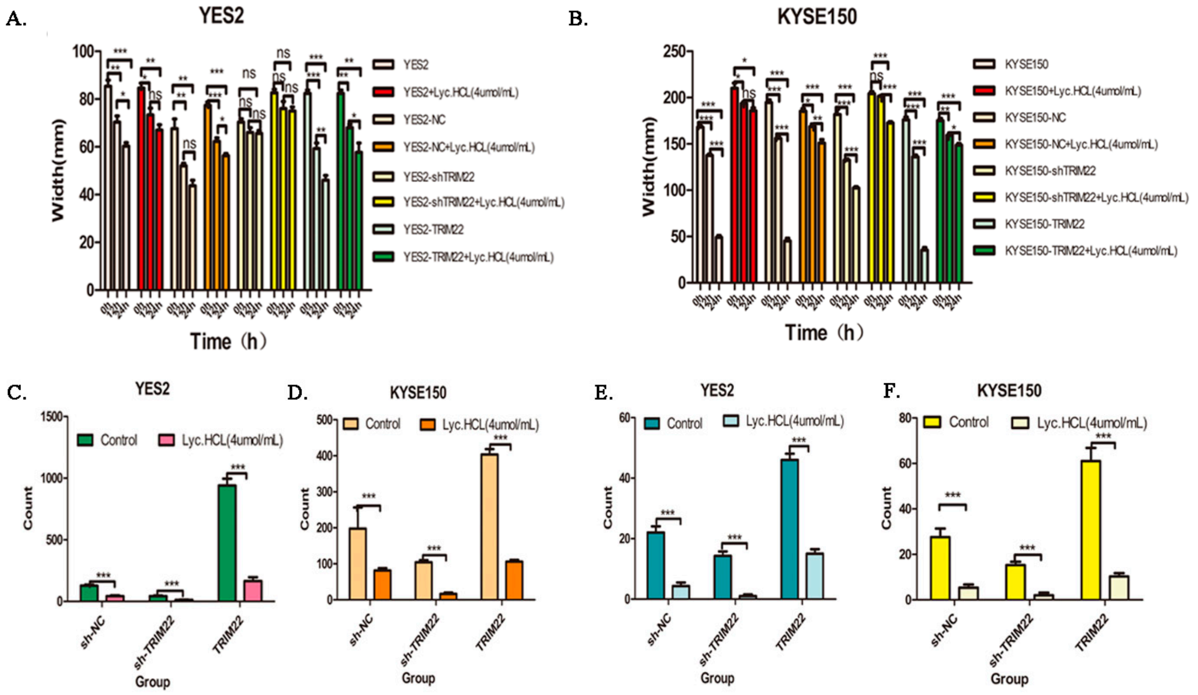

Supplementary Figure S7. Data analysis of Figure 7. (A and B) Gap Width data of Figure 7A and 7B were presented as the mean ± SD. A one-way analysis of variance, followed by a Tukey's post-hoc test, was used to compare the different groups. *P<0.05, **P<0.01, ***P<0.001 versus vehicle. (C and D) Quantification of the migrated cells in Figure 7C and 7D were displayed. The results were displayed as the mean ± SD. A one-way analysis of variance, followed by a Tukey's post-hoc test, was used to compare the different groups. *P<0.05, **P<0.01, ***P<0.001 versus vehicle. (E and F) Quantification of the invasive cells in Figure 7E and 7F were shown. The results are presented as the mean ± SD. A one-way analysis of variance (ANOVA), followed by Tukey's post-hoc test, was used to compare the different groups. *P < 0.05, **P < 0.01, ***P < 0.001 versus vehicle.

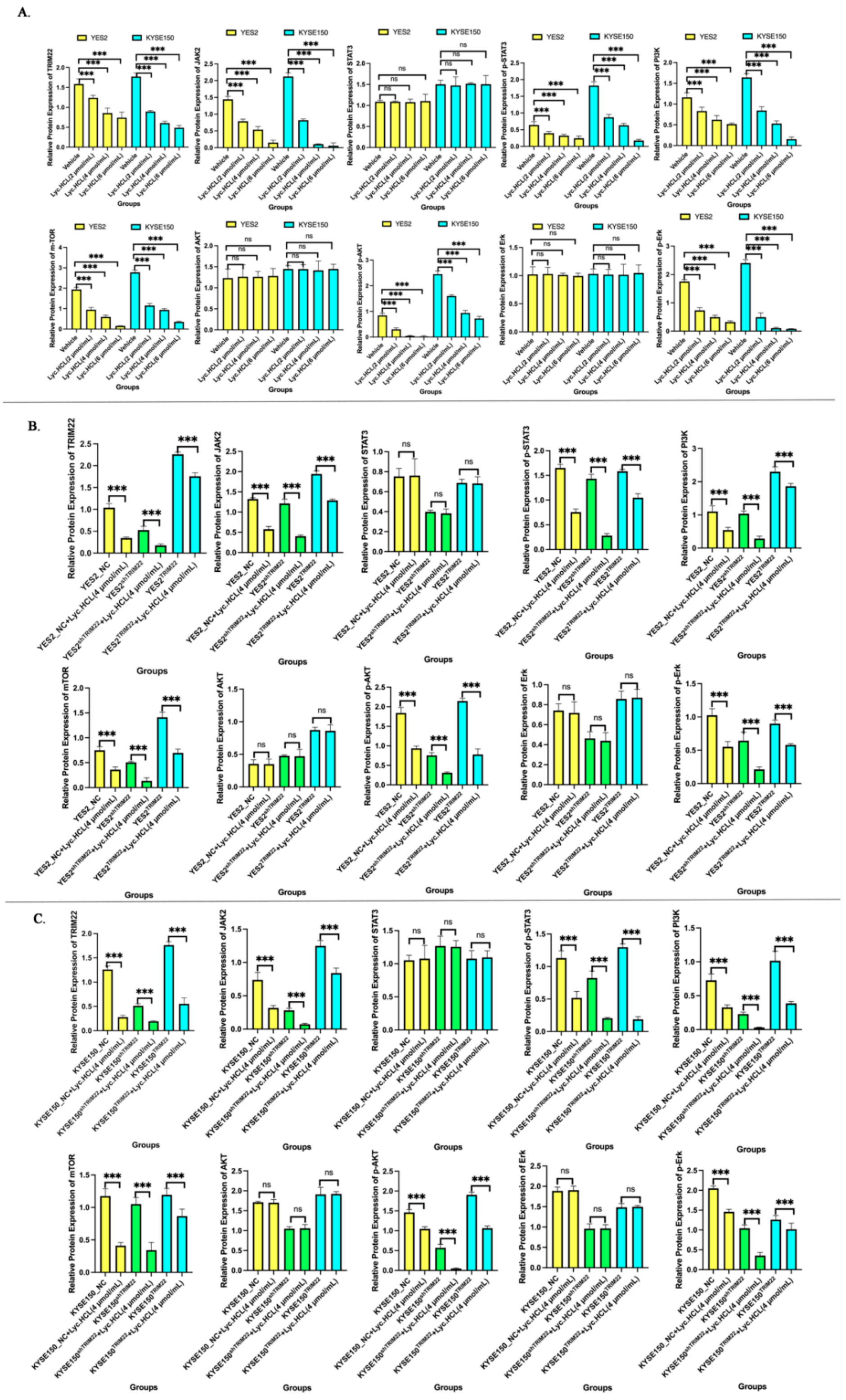

Supplementary Figure S8. Data analysis of Figure 8. (A) Protein quantification of relative expression levels in Figure 8A were performed using ImageJ. (B) Protein quantification of relative expression levels in Figure 8B were

performed using ImageJ. (C) Protein quantification of relative expression levels in Figure 8C were performed using ImageJ.

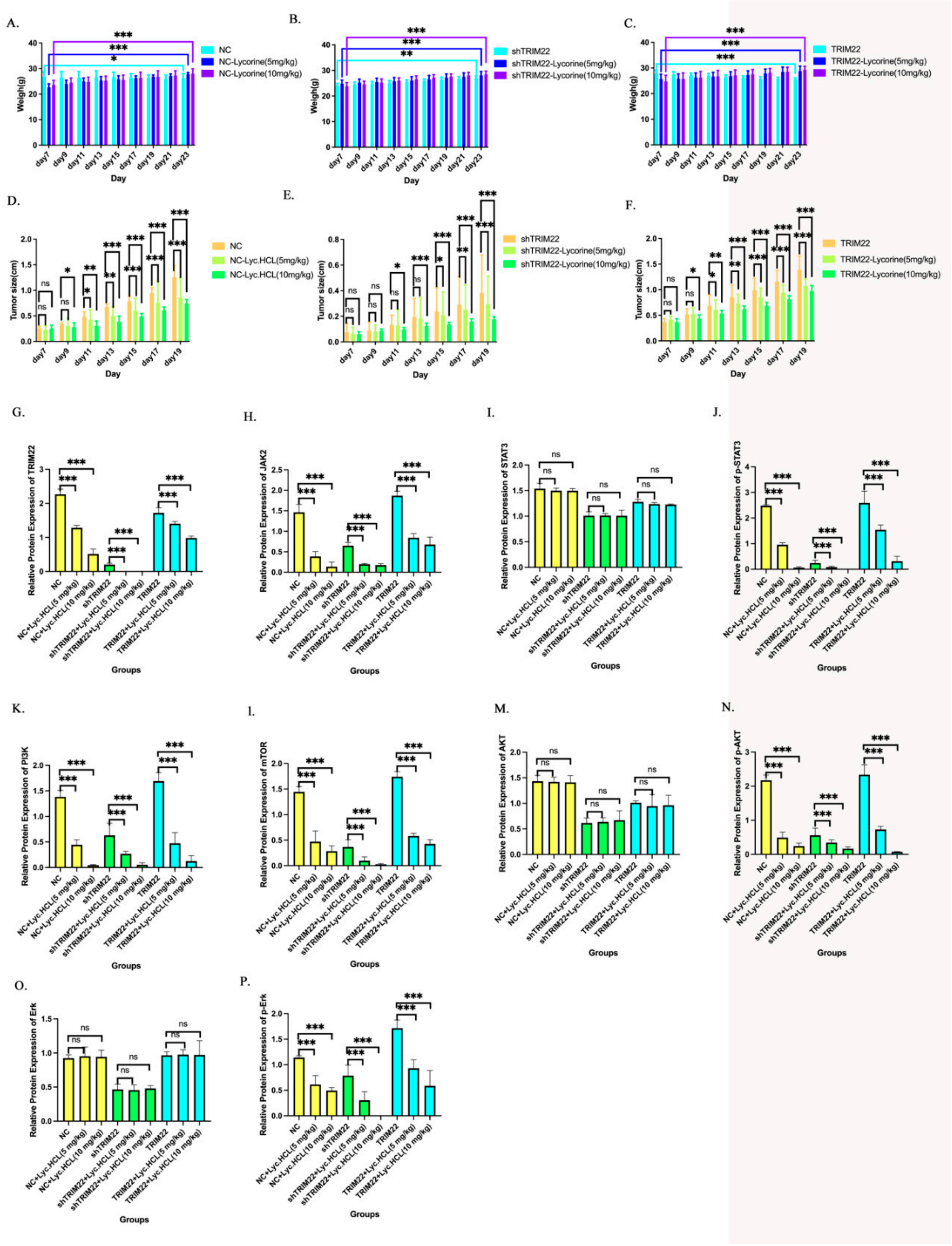

Supplementary Figure S9. Data analysis of Figure 10. (A-C) Analysis of the body weight changes of the mice. (D-F) Tumor size analysis. (G-P) Quantification of the relative protein expression of Figure 10D.
